# Marginal leakage of retrograde filling materials using stereomicroscope: An in vitro study

**DOI:** 10.6026/973206300210394

**Published:** 2025-03-31

**Authors:** Abdullah Abdullatif Mahdi, Debjit Dhamali, Krishnan Hari, Dinesh Kamath, Elizabeth Issac, Varsha Sam, Anzil K.S Ali

**Affiliations:** 1 Department of Restorative and Dental implants, Allcare Medical Center, Abu Dhabi, UAE; 2 Department of Dentistry, IQCITY Medical College and Hospital, Durgapur, West Bengal, India; 3 Department of Conservative Dentistry and Endodontics, Mar Baselios Dental College, Kothamangalam, Kerala, India; 4 Department of Conservative Dentistry and Endodontics, Indira Gandhi Institute of Dental Sciences, Kothamangalam, Kerala, India; 5 Department of Conservative Dentistry and Endodontics, Travancore Dental College, Kollam, Kerala, India; 6 Department of Public Health Dentistry, Royal Dental College, Palakkad, Kerala, India

**Keywords:** Apical microleakage, biodentine, calcium silicate cement, retrograde filling, root repair material

## Abstract

Root-end filling material apical microleakage in ultrasonic retro tip-prepared retro-cavities is of interest to dentists. Hence, 68 entire maxillary second premolars and mandibular premolars with a single root, removed for orthodontic reasons from individuals were selected for this study. A 3 mm apical root-end excision was performed utilizing a diamond disc while, root-end cavities were created utilizing an ultrasonic retro-tip. Four groups of 17 teeth were randomly assigned to receive retrograde cavity repairs with mineral trioxide aggregate (group 1); Biodentine (group 2), total fill bioceramic root repair material (group 3) and resin-modified glass ionomer cement (group 4). It was observed that bioceramic root repair material (0.197±0.341), biodentine (0.256±0.547) and mineral trioxide aggregate (0.814±0.436) exhibited a significantly lesser microleakage than resin-modified glass ionomer cement (1.381±0.743). Thus, the bioceramic root repair material exhibited the least mean microleakage among all the materials that were assessed in this study.

## Background:

The basic prerequisite for successful endodontic therapy is the thorough obliteration of the root canal and the establishment of a fluid-tight seal [1]. The successful therapy of periapical pathogenesis is not attained in certain instances, despite the emergence of improved materials and instruments and the implementation of novel endodontic strategies [2]. When traditional endodontic treatment fails, surgical endodontic surgery is required to preserve the affected tooth [1]. This treatment entails exposing the affected apex, removal of the apical root-end, preparing the root-end cavity and insertion of root-end filling material [3]. The optimal root-end filler material must be easily manipulated, radiopaque, stable in dimension, nonabsorbable and impervious to moisture. It must conform to the preparation walls, seal the root canals, be biocompatible and facilitate healing. A multitude of root-end filling materials exists, such as zinc oxide-eugenol (ZnOE), reinforced ZnOE, amalgam, gutta-percha, composite resin, mineral trioxide aggregate, zinc phosphate and carboxylate cement and gold foil [4, 5]. In recent years, several novel bioceramic materials such as Bio Aggregate, Biodentine and Endosequence root repair material (RRM) have been introduced to the market [1]. Glass ionomer cement possesses universal characteristics. It serves as a dentin substitute, demonstrating the capacity to form chemical bonds with tooth structure, so ensuring an exceptional marginal seal. Research indicates that glass ionomer cement exhibits antibacterial properties owing to the gradual dissolution of fluorides. Nonetheless, the marginal seal is undermined due to its breakdown in biological fluids and its susceptibility to technique sensitivity [4, 5- 6]. The physical characteristics of the conventional glass ionomer cement are improved by the resin-modified glass ionomer cement, which also reduces their sensitivity to water balance. It also has a longer working time, enhanced translucency, a more rapid set and the ability to achieve early strength [7, 8]. Calcium silicate cement materials, commonly referred to as Bioceramic, whether in the form of a sealer or a thicker combination, are regarded as the optimal endodontic material for retrograde therapy owing to their superior physicochemical and biological features, particularly biocompatibility and stability. This inorganic, non-corrosive ceramic cement comprise tricalcium silicate and other radiopaque particles [9]. Mineral trioxide aggregate, calcium silicate cements and serves as a retrograde material with superior adaptability to cavity walls, commendable biocompatibility and minimal solubility [10]. Sealed lateral canals inhibit the further contamination of the endodontic system and discharge calcium ions, facilitating fast tissue repair [11]. Total Fill bioceramic root repair material is a reformulated substance in a putty or syringe form. The primary constituents are calcium silicate and zirconium oxide and demonstrate an enhanced healing of periradicular tissues. It displays enhanced handling qualities and sets quickly. Furthermore, its biocompatibility is comparable to mineral trioxide aggregate [3]. Biodentine is calcium silicate cement with superior strength comparable to mineral trioxide aggregate, exhibiting enhanced physicochemical properties such as a reduced setting time and elevated mechanical strength, facilitating its clinical application in both traditional root canal therapy and restorative cases involving dentine substitutes [5, 6, 7, 8- 9]. Apical microleakage refers to the seepage occurring at the junction between the filling materials and the canal wall [12]. Apical microleakage is a subject of research due to the persistent occurrence of clinical failure, despite advancements in endodontics. An inadequate marginal seal of the retrocavity might permit the infiltration of microbes and byproducts into the root canal and periradicular structures, potentially failing therapy [1]. Apical sealing achieved with retrograde filling materials can be assessed through dye penetration depth, fluid-filtration techniques, radioisotope or bacterial infiltration, or electrochemical procedures. The dye penetration approach is the predominant and readily executed technique [13- 14]. Therefore, it is of interest to compare the apical microleakage of root-end filling materials such as mineral trioxide aggregate, biodentine, bioceramic root repair material and resin-modified glass ionomer cement in cavities that have been prepared using ultrasonic retro tips.

## Methods and Materials:

The study employed G*Power 3.1.9.7 (Heinrich-Heine-Universität, Germany) to determine an optimal sample size of 68 teeth, utilizing a statistical power of 96%, at a 5% significance level and an effect size of 0.53 [14], then randomized them into four groups, each including 17 teeth. A sample of 68 intact maxillary second premolars and mandibular premolars with a single root, removed for orthodontic reasons from individuals of comparable age, with a closed apex, caries-free status, similar size, no evidence of fractures/cracks and an identical root length, was chosen. The teeth were examined using a stereomicroscope (Carl Zeiss, Jena, Germany) to assess the number of canals, cracks and dental caries. Teeth having multiple roots, additional canals, open apices, root caries and calcified roots were eliminated. The chosen teeth were submerged in a 5% sodium hypochlorite solution (Prevest Den Pro, India) for five minutes. An ultrasonic scaler was employed to eliminate debris, calculus and soft tissues and the teeth were thereafter preserved in a vase with a 10% buffered formalin solution for future usage. The coronal portion of the teeth was horizontally divided along the longitudinal axis using a diamond disc at the cementoenamel junction level or further down, to standardize the root length to 15 mm. A preoperative radiograph was taken and an access opening was established using an access bur (Dentsply Maillefer, USA). The radiographic working length determination was carried out using#10 K-file, while a #40 K-file served as the master apical file (Mani *Inc.*, Japan). Ethylenediaminetetraacetic acid (EDTA) irrigation was administered initially, after which was irrigated with 5% sodium hypochlorite and concluded with saline irrigation. Drying and obturation were done using lateral compaction, 2% gutta-percha cones (Meta Biomed, Korea) and AH Plus (Dentsply Maillefer, Switzerland) root canal sealant. Obturation was followed by composite resin filling. The specimens were saline-immersed for a week and were kept in a 535-liter Binder incubator (Tuttlingen, Germany) with a 0.58 m^2^ footprint, at 37°C and 100% humidity for five days. The samples were then dissected apically at 90° to the longitudinal root axis using a cross-cut fissure bur to remove 3 mm of the apex. Root-end conventional retrocavity was prepared with an ultrasonic retro-tip (Satelac in P5 Satelac unit at medium power setting) to 3 mm depth. Four groups of 17 teeth were randomly assigned to receive retrograde cavity repairs with mineral trioxide aggregate; biodentine, bioceramic root repair material and resin-modified glass ionomer cement which were designated as groups 1 through 4 respectively. The materials were processed following the manufacturer's specifications, thereafter restoring the retrocavity. The specimens were maintained at 100% humidity and 37°C for five days. The established retrocavities were subjected to washing, saline irrigation and drying. Subsequently, the specimens had been painted with nail varnish, leaving the apical one mm uncoated and allowed to dry. The teeth were immersed in 1% methylene blue (MB) dye for 48 hours. The root surfaces were cleansed and divided along the longitudinal axis with a diamond disc utilizing water cooling. Dye permeation was analyzed using a stereomicroscope and grading was conducted on a score from 0 to 3. The extent of dye infiltration was determined following the criteria of Mandava *et al.* [15] as follows: 0 denotes the absence of penetration; 1 indicates penetration into the enamel or cementum surface of the preparation wall; 2 represents penetration into the dentin portion of the preparation wall, excluding the pulpal floor; and 3 denotes penetration encompassing the pulpal bottom of the preparation.

## Statistical analysis:

IBM SPSS Statistics for Windows, Version 26.0 (IBM Corp., Armonk, New York, USA) was employed to analyze the data. The data was subjected to one-way ANOVA and Tukey's post hoc multiple comparison test. The significance of the variation in the extent of dye infiltration between the groups was determined using the Chi-square test.

## Results:

The statistical significance of the overall comparison of mean microleakage values across the groups is illustrated in Table 1. The bioceramic root repair material (group 3: 0.197±0.341) specimen exhibited the lowest mean microleakage, subsequently followed by the biodentine (group 2: 0.256±0.547), mineral trioxide aggregate (group 1: 0.814±0.436) and resin-modified glass ionomer cement (group 4: 1.381±0.743) specimens. The results of the multiple comparisons suggest that the microleakages in Groups 2 and 3 samples were statistically insignificant (p=0.71) suggesting that the microleakage between the biodentine and bioceramic root repair material groups was identical. Conversely, the microleakages of the remaining groups were statistically significantly different in the pair-wise comparison (p<0.01). It was determined that the extent of dye penetration among the study groups was statistically highly significant (p<0.01). The resin-modified glass ionomer cement specimen reported a higher frequency (35.3%) of score 3 (dye penetration into the pulpal floor of the preparation), while the bioceramic root repair material group (52.9%) observed no leakage (score 0) more frequently (Table 2).

## Discussion:

Among the materials employed for retrograde filling in the present investigation, bioceramic root repair material (Group 3) demonstrated the least microleakage, succeeded by biodentine (Group 2), mineral trioxide aggregate (Group 1) and resin-modified glass ionomer cement (Group 4). The findings of the current investigation concurred with those of Angitha *et al.* [14] The calcium phosphate monobasic in bioceramic root repair material contains an additive that promotes hydroxyapatite production and exhibits characteristics that involve wear resistance, biological compatibility, chemical longevity and aesthetic appeal [16]. Biodentine demonstrated microleakage levels identical to those of bioceramic root repair material in the current investigation owing to the reduced setting time of 12 minutes and the generation of tag-like structures consisting of calcium or phosphate-rich crystalline deposition between the tooth and root-end filling materials [17]. This aligns with the research conducted by Kokate and Pawar [5], Singh *et al.* [18] and Nanjappa *et al.* [17], which demonstrated that biodentine exhibited superior sealing capability. The work by Mandava *et al.* [15] assessed the apical microleakage of root-end cavities that were filled with mineral trioxide aggregate, biodentine and light-activated glass ionomer cement, utilizing distinct cavity preparation methods namely standard bur and ultrasonic tip preparations. The findings of their investigation indicated markedly reduced microleakage of mineral trioxide aggregate in comparison to biodentine and light-activated glass ionomer cement, which contradicts our data. Periradicular surgery involves removing damaged Periradicular tissue, root-end excision, retrocavity preparation and root canal filling [19]. The findings of our investigation concur with prior research indicating that mineral trioxide aggregate exhibits superior marginal sealing compared to alternative retrograde fillers, including glass ionomer cement, light-activated glass ionomer cement and amalgam [20]. This may be attributed to the development of hydroxyapatite-like crystals at the interfaces of the material and root canal dentine, which facilitates superior adhesion and inhibits dye penetration [21]. Biodentine has superior properties compared to mineral trioxide aggregate due to its expedited setting time, hence diminishing the possibility of bacterial infiltration [22]. Biodentine demonstrates enhanced sealing capabilities compared to mineral trioxide aggregate [23]. Furthermore, it demonstrates superior biomineralization compared to mineral trioxide aggregate, resulting in a more extensive development of calcium-rich layers [14]. Radeva *et al.* [24], Naik *et al.* [25] and Khandelwal *et al.* [26] corroborated the findings of this investigation, concluding that biodentine exhibits superior sealing capability compared to mineral trioxide aggregate. The linear permeation of 1% MB dye was quantified in the current investigation. MB is frequently utilized due to its low molecular weight, which enhances its penetrability [27]. Methylene blue dyes have been utilized frequently to evaluate the sealing ability of root-end filling materials [28].

In several other experiments [15-29], Rhodamine B, a water-soluble fluorescent dye, was employed. It is readily identifiable, even at low concentrations, migrates easily along the interface, has minimal toxic effects and remains stable in aqueous environments and across different pH levels, while being non-invasive to the substrate or component in contact. Lucena-Martin *et al.* [30] demonstrated that the transverse root sectioning technique leads to a breakdown of dye and dentin material. Thus, the longitudinal sectioning technique assessed dye infiltration into filler materials. Ultrasonic retro-tips outperform traditional burs for retrocavity preparation. Applying ultrasonic tips for preparing the root-end cavity results in minimal alteration to the root canal architecture and is accurate and more hygienic [14]. Nonetheless, a disadvantage of employing ultrasonics is the formation of microcracks on the walls of root canals. The production of cracks is correlated with the intensity of the ultrasonic equipment; therefore, a lower intensity is advisable [31]. No damage to dentine tissues is evident at the low-level intensity of 4 MHz utilized in this study, consistent with the findings of Khandelwal *et al.* [26]. This is corroborated by the inability to identify cracks under SEM in prior research [32]. The angle of root excision is a critical concern. The presence of open dentinal tubules may compromise the healing of the lesion as a consequence of inclined plane sectioning 33]. Therefore, the cutting blade is oriented at 90° to the longitudinal root axis, thereby decreasing the quantity of patent dentinal tubules at the open end and mitigating microleakage [34]. Moreover, excision to a depth of 3 mm diminishes apical ramifications by 98% and lateral canals by 93% [5]. The cavities created with a conventional bur using a slow-speed hand-piece generate significant quantities of smear layer in contrast to ultrasonic tips. This debris is permeable to toxic substances, thereby inhibiting direct contact between the material and the cavity walls which could explain the increased microleakage observed in retro-cavities created with traditional burs in slow-speed hand-pieces. It was suggested that when employing a material that fails to establish a hermetic seal, the preparation of the cavity with diamond-coated ultrasonic tips is recommended to enhance the seal and marginal fit [32]. A confocal laser scanning microscope, a non-invasive technique for visualizing dye permeation, can be utilized as opposed to a stereomicroscope. This method offers specific advantages in visualizing subsurface cell characteristics, including a distinct delineation of leakage boundaries, attributable to lens focus occurred several microns below the visible surface [30]. It aids in preventing stain diffusion resulting from specimen sectioning and diminishes polishing artifacts that may enhance the depth of dye penetration [35]. It additionally prevents the dispersed, reflected and fluorescent light from many planes and enhances clarity in the focal axis [36]. Moreover, since In vitro assessments do not consistently reflect in vivo efficacy, clinical trials are necessary to enhance the significance of outcomes.

## Conclusion:

It was observed that bioceramic root repair material (BC-RRM), biodentine and mineral trioxide aggregate exhibited a significantly lesser microleakage than resin-modified glass ionomer cement. The bioceramic root repair material showed the least mean microleakage among all the materials that were assessed. The bioceramic root repair material group had more no-leakage (score 0) than the resin-modified glass ionomer cement specimen, which had more score of 3 (dye penetration into the pulpal floor).

## Figures and Tables

**Table 1:** Comparison of mean microleakage values among the study groups

**N=17/group**	**Mean±SD**	**ANOVA**		**Tukey's post hoc test**					
		**F-test**	**p-value**	**1 Vs 2**	**1 Vs 3**	**1 Vs 4**	**2 Vs 3**	**2 Vs 4**	**3 Vs 4**
Group 1	0.814±0.436	18.036	0.000**	0.002**	0.000**	0.01**	0.71	0.000**	0.000**
Group 2	0.256±0.547								
Group 3	0.197±0.341								
Group 4	1.381±0.743								
**Highly significant;
p<0.05 - not significant

**Table 2:** Comparison of the extent of dye penetration among the study groups

**Groups**	**N**	**Dye penetration scores**				**Fisher's exact test**	**p-value**
		**0 N(%)**	**1 N(%)**	**2 N(%)**	**3 N(%)**		
Group 1	17	4(23.5%)	7(41.2%)	5(29.4%)	1(5.9%)	28.01	0.001**
Group 2	17	6(35.3%)	8(47.1%)	3(17.6%)	0(0)		
Group 3	17	9(52.9%)	6(35.3%)	2(11.8%)	0(0)		
Group 4	17	0(0)	4(23.5%)	7(41.2%)	6(35.3%)		
**Highly significant
